# Enteroscopy versus Video Capsule Endoscopy for Automatic Diagnosis of Small Bowel Disorders—A Comparative Analysis of Artificial Intelligence Applications

**DOI:** 10.3390/biomedicines11112991

**Published:** 2023-11-07

**Authors:** Stefan Lucian Popa, Bogdan Stancu, Abdulrahman Ismaiel, Daria Claudia Turtoi, Vlad Dumitru Brata, Traian Adrian Duse, Roxana Bolchis, Alexandru Marius Padureanu, Miruna Oana Dita, Atamyrat Bashimov, Victor Incze, Edoardo Pinna, Simona Grad, Andrei-Vasile Pop, Dinu Iuliu Dumitrascu, Mihai Alexandru Munteanu, Teodora Surdea-Blaga, Florin Vasile Mihaileanu

**Affiliations:** 1 2nd Medical Department, “Iuliu Hatieganu” University of Medicine and Pharmacy, 400000 Cluj-Napoca, Romania; popa.stefan@umfcluj.ro (S.L.P.); abdulrahman.ismaiel@yahoo.com (A.I.); costinsimona_m@yahoo.com (S.G.); andreipopdr@gmail.com (A.-V.P.); dora_blaga@yahoo.com (T.S.-B.); 2 2nd Surgical Department, “Iuliu Hatieganu” University of Medicine and Pharmacy, 400347 Cluj-Napoca, Romania; ms26rfl@yahoo.com; 3 Faculty of Medicine, “Iuliu Hatieganu“ University of Medicine and Pharmacy, 400000 Cluj-Napoca, Romania; turtoidariaclaudia@gmail.com (D.C.T.); brata_vlad@yahoo.com (V.D.B.); adrianduse@yahoo.com (T.A.D.); bolchis.roxana@yahoo.com (R.B.); alexandru.padureanu@outlook.com (A.M.P.); miruna.dita@outlook.com (M.O.D.); atamyrat.tm.98@gmail.com (A.B.); vicincze@yahoo.com (V.I.); edoardo.pinna9210@gmail.com (E.P.); 4 Department of Anatomy, “Iuliu Hatieganu“ University of Medicine and Pharmacy, 400006 Cluj-Napoca, Romania; d.dumitrascu@yahoo.com; 5 Department of Medical Disciplines, Faculty of Medicine and Pharmacy, University of Oradea, 410087 Oradea, Romania; mihaimunteanual@yahoo.com

**Keywords:** small bowel, artificial intelligence, enteroscopy, video capsule endoscopy

## Abstract

Background: Small bowel disorders present a diagnostic challenge due to the limited accessibility of the small intestine. Accurate diagnosis is made with the aid of specific procedures, like capsule endoscopy or double-ballon enteroscopy, but they are not usually solicited and not widely accessible. This study aims to assess and compare the diagnostic effectiveness of enteroscopy and video capsule endoscopy (VCE) when combined with artificial intelligence (AI) algorithms for the automatic detection of small bowel diseases. Materials and methods: We performed an extensive literature search for relevant studies about AI applications capable of identifying small bowel disorders using enteroscopy and VCE, published between 2012 and 2023, employing PubMed, Cochrane Library, Google Scholar, Embase, Scopus, and ClinicalTrials.gov databases. Results: Our investigation discovered a total of 27 publications, out of which 21 studies assessed the application of VCE, while the remaining 6 articles analyzed the enteroscopy procedure. The included studies portrayed that both investigations, enhanced by AI, exhibited a high level of diagnostic accuracy. Enteroscopy demonstrated superior diagnostic capability, providing precise identification of small bowel pathologies with the added advantage of enabling immediate therapeutic intervention. The choice between these modalities should be guided by clinical context, patient preference, and resource availability. Studies with larger sample sizes and prospective designs are warranted to validate these results and optimize the integration of AI in small bowel diagnostics. Conclusions: The current analysis demonstrates that both enteroscopy and VCE with AI augmentation exhibit comparable diagnostic performance for the automatic detection of small bowel disorders.

## 1. Introduction

Small bowel disorders pose diagnostic challenges due to their location, limited symptoms, and the lack of routine screening. Situated deep within the abdomen, the small bowel is not easily accessible for direct examination. Moreover, its symptoms—abdominal pain, bloating, diarrhea, and weight loss—are non-specific and can mimic various gastrointestinal conditions [1,2,3]. Traditional imaging techniques often fail to visualize this coiled organ adequately, and routine endoscopic procedures can only access its ends, leaving a significant portion unexamined. Specialized tests like capsule endoscopy or double balloon enteroscopy are required to diagnose small bowel disorders. However, these are not as widely available and may not be routinely considered unless specifically indicated [4]. Additionally, the transit time of substances through the small bowel can vary, making it challenging to identify abnormalities based on transit time alone [5]. Furthermore, some small bowel disorders are rare and may not be initially considered during the diagnostic process, leading to potential delays or missed diagnoses. These factors collectively contribute to the complexity and difficulty of diagnosing small bowel disorders.

Cancers of the small bowel, specifically small intestine cancer, can also be challenging to diagnose. These cancers are relatively rare compared to other digestive organ tumors, and their symptoms can mimic various benign gastrointestinal conditions, making them prone to misdiagnosis [6]. Common symptoms of small intestine cancer include abdominal pain, unexplained weight loss, changes in bowel habits (diarrhea or constipation), blood in the stool, and abdominal bloating [7]. These non-specific symptoms can be attributed to many other digestive disorders, including inflammatory conditions, irritable bowel syndrome (IBS), or even more common malignancies like colorectal cancer. A combination of imaging studies (CT scans, MRIs), endoscopic procedures, and tissue biopsies is typically required to diagnose small intestine cancer. Due to the difficulty in diagnosing small bowel cancers, healthcare providers may need to maintain a high level of suspicion when patients present with persistent, unexplained gastrointestinal symptoms to ensure timely and accurate diagnosis and treatment [6,8].

Capsule endoscopy and enteroscopy represent two endoscopic techniques with diagnostic utility regarding small bowel disorders. While capsule endoscopy has been frequently used since 2000, allowing for a simple and comprehensive evaluation of patients suspected of small bowel disorders, certain disadvantages come with its non-invasiveness. Therefore, device-assisted enteroscopy (DAE) comes as an essential step after a capsule endoscopy investigation reveals abnormalities, an indication for therapeutic intervention, or no certain diagnosis can be established [9].

Artificial intelligence (AI) holds immense promise for the future of precise diagnosis in small bowel disorders due to its capacity to harness complex data analysis, image recognition, and pattern recognition algorithms at a scale and speed far surpassing human capabilities [10]. In the context of small bowel disorders, AI can process and interpret vast amounts of medical imaging data, including capsule endoscopy and radiological scans, with remarkable accuracy, swiftly identifying subtle abnormalities or lesions that may elude human detection. Machine learning algorithms can assimilate diverse patient data, such as clinical history, genetics, and biomarkers, to refine diagnostic accuracy and predict disease progression, enabling tailored treatment strategies [10]. Furthermore, AI can assist in differential diagnosis, distinguishing small bowel disorders from phenotypically similar conditions like IBS or inflammatory bowel disease, reducing the likelihood of misdiagnosis. Moreover, AI-powered predictive modelling can facilitate early disease identification, potentially enabling interventions before severe complications manifest. While the application of AI in small bowel disorder diagnosis is still evolving, its integration into clinical practice has the potential to revolutionize healthcare by enhancing diagnostic precision, expediting therapeutic interventions, and ultimately improving patient outcomes in this challenging diagnostic realm.

For this reason, we conducted a comparative analysis of AI applications capable of automatically diagnosing small bowel disorders. We assessed the accuracy of enteroscopy versus video capsule endoscopy. Our research meticulously scrutinized the diagnostic accuracy achieved by AI models in discerning a spectrum of small bowel disorders, including entities such as Crohn’s disease, ulcers, neoplastic lesions, and vascular abnormalities.

Moreover, this comparative analysis elucidates the practical implications of implementing AI-facilitated diagnostic tools within authentic clinical scenarios. It outlines potential advantages, including reduced procedural duration, enhanced patient comfort, and increased diagnostic yield. Additionally, it evaluates the constraints inherent to both enteroscopy and VCE, as well as the AI models themselves. Factors such as economic considerations, accessibility, and the need for expert oversight are thoughtfully considered.

This study provides valuable insights into the advances in artificial intelligence’s potential utility in diagnosing small bowel disorders. By scrutinizing the performance of AI models in conjunction with enteroscopy and VCE, the research offers invaluable information that holds the promise of significantly impacting clinical practice and patient care within the gastroenterological field.

## 2. Materials and Methods

This systematic review has been conducted in accordance with the Preferred Reporting Items for Systematic Reviews and Meta-analyses reporting guidelines (PRISMA) [11].

Search strategy

A comprehensive literature search was conducted from 2012 until 2023, using Pubmed, MEDLINE, Cochrane Library, Google Scholar, Embase, and ClinicalTrials.gov databases. Keywords used in the search strategy included “Artificial Intelligence”, “Machine Learning”, “Deep Learning”, “Convolutional Neural Network (CNN)”, “Small Bowel Disorders”, “Capsule Endoscopy”, “Enteroscopy”, “Diagnosis”, “Imaging”, “Image Analysis”, and “Computer-Aided Diagnosis” in various combinations, with the help of Boolean operators (AND, OR, NOT). Searches were also performed using Medical Subject Headings (MeSH) where applicable. Language restrictions to English, Romanian, and German were applied.

Eligibility criteria

The eligibility criteria were based on the PICOS framework to select the relevant literature.

- Population: Studies involving human subjects of all ages diagnosed with or suspected small bowel disorders have been included, whereas studies involving animal subjects or in vitro have been excluded.
- Intervention: Studies that utilize artificial intelligence (AI), machine learning, deep learning, convolutional neural networks, or computer-aided diagnosis in the processing of capsule endoscopy and/or enteroscopy images for diagnosis were added. Studies using capsule endoscopy or enteroscopy without any form of AI for diagnosis or other gastrointestinal diseases without focusing on small bowel disorders were removed.
- Comparator: The presence of a control group was not mandatory for the screening and selection process. If a control group was present, it had to be diagnosed through standard diagnostic methods without the use of AI.
- Outcome Measures: Studies should focus on diagnostic metrics such as sensitivity, specificity, predictive values, or other performance metrics of AI-based techniques and not patient satisfaction or cost-effectiveness, which offers no objective performance measurement.

Study Design

Peer-reviewed original research articles, including randomized controlled trials, cohort studies, and case–control studies. Reviews, letters to the editor, commentaries, case reports, case series, and animal studies were excluded.

Studies with incomplete data sets or lacking the necessary statistical analysis and duplicate publications, where multiple articles based on the same dataset were excluded, keeping only the most comprehensive one.

Selection and data extraction

A standardized data extraction form has been developed and pilot-tested on a subset of studies to ensure effectiveness. The form included study identifiers (e.g., authors, year of publication), study design (e.g., RCT, cohort study), study population (e.g., sample size, demographics), interventions, and outcome measures.

**Figure 1:**
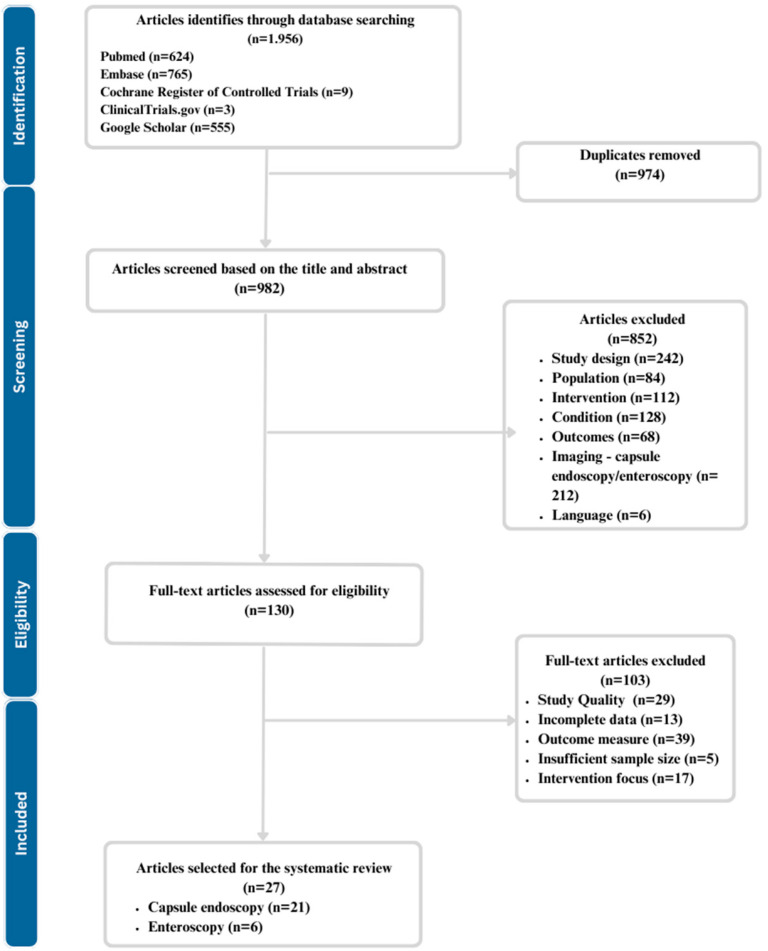
PRISMA flow diagram for study selection.

The process encompasses more steps and is visually represented in Figure 1.

- Initial Screening: The title and abstract have been screened for relevance by 2 independent reviewers. Each database was progressively, one at a time/one after the other screened for title and abstract. All relevant articles have been added to the Mendeley Account in the folder dedicated to abstract and title screening. Afterwards, the duplicates were removed with a function integrated into Mendeley.
- Full-Text Review for Eligibility: For studies passing the initial screening and entering the eligibility stage, full-text articles were retrieved and assessed for eligibility based on predefined inclusion and exclusion criteria. Inclusion criteria included studies that applied AI to either enteroscopy or capsule endoscopy for diagnosing small bowel disorders and provided data on diagnostic accuracy. Exclusion criteria included case reports, conference abstracts, non-English articles, studies lacking relevant data, and others elaborated in the eligibility criteria section.
- Data Extraction: Data were extracted using a standardized form by two independent reviewers. The extracted information included study design, sample size, type of AI algorithm, small bowel disorder investigated, diagnostic modality (enteroscopy or capsule endoscopy), diagnostic performance metrics (such as sensitivity, specificity, accuracy), and any usability measures (e.g., time efficiency), using the standardized form. Discrepancies in data extraction were resolved through discussion and consensus.

Any discrepancies between reviewers during the data extraction have been resolved through discussion. Missing data has been requested from study authors where feasible.

Data Management

All extracted data has been entered into a database with restricted access to ensure data integrity. The database has been backed up regularly.

Quality assessment

**Figure 2:**
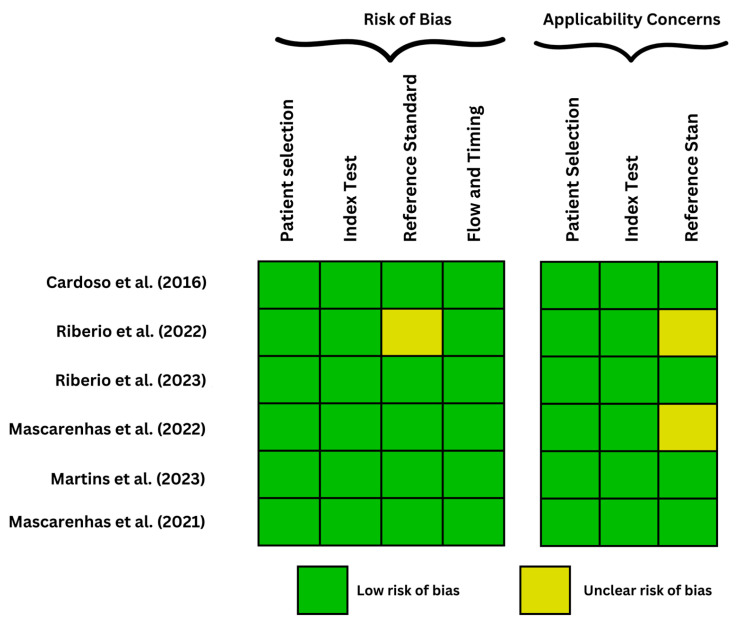
QUADAS-2 framework evaluating the risk of bias of studies focused on capsule endoscopy [12,13,14,15,16,17].

**Figure 3:**
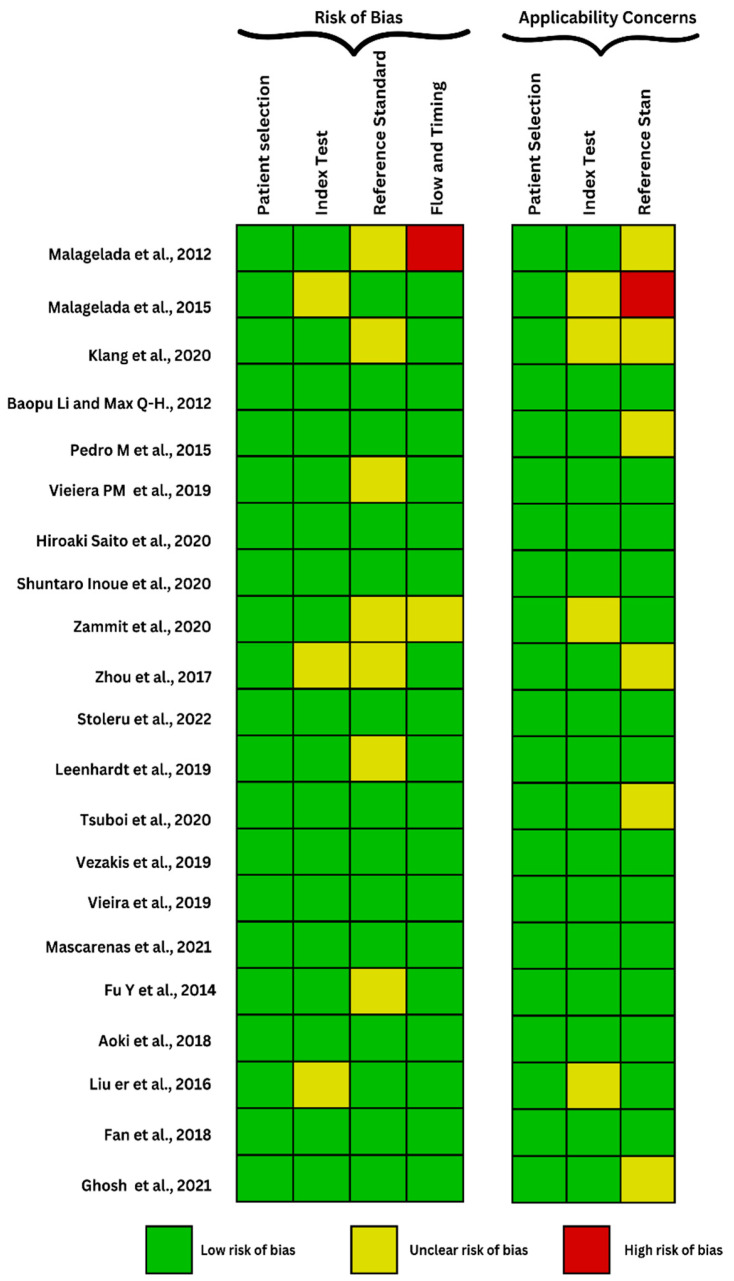
QUADAS-2 framework evaluating the risk of bias of studies focused enteroscopy [18,19,20,21,22,23,24,25,26,27,28,29,30,31,32,33,34,35,36,37,38].

The quality of the included studies was assessed using the QUADAS-2 tool, as seen in Figure 2 and Figure 3, evaluating the risk of bias and applicability concerns in diagnostic accuracy studies. It primarily consists of four key domains: patient selection, index test, reference standard, and flow and timing utilized in 4 phases with signaling tasks and question for bias risk awareness. Two reviewers independently performed the assessment, and any disagreements were resolved through consensus or consultation with a third reviewer.

## 3. Results

A summary of the descriptive characteristics and some of the evaluation metrics of the included studies are presented in Table 1.

### 3.1. Videocapsule Endoscopy

Capsule endoscopy is currently being used to diagnose many small bowel conditions. AI has been shown as a diagnostic aid in several small bowel diseases, such as functional small bowel disorders, Chron’s disease, malignant abnormalities, celiac disease, angioectasia, and erosions and ulcers of various etiology.

Functional small bowel disorders are currently diagnosed based on the Rome IV criteria, which entails symptom analysis items [39]. Unfortunately, clinical diagnostic criteria do not correspond to the pathophysiological mechanisms in the small bowel, thus leading to treatment failure and persistence of the symptoms, prolonging the precarious life quality of the patients [40].

Malagelada et al. conducted two studies intending to introduce a novel, objective classification model of small bowel functional disorders based on dysmotility patterns [18,19]. Using computer vision for image computation, the machine learning (ML) algorithm, a support vector machine (SVM) detected several significantly different indexes (19 in the first study and 43 in the second study) in healthy subjects versus functional disorder patients. These were used to discern between motility, contractile and non-contractile patterns, and endoluminal content in terms of frequency and duration, with an additional grouping into hypomotility and hypermotility behaviors.

The first study had a cohort consisting of only 80 patients with functional disorders and 70 healthy patients, and the same population was used for training and testing the SVM [18]. The subsequent prospective study improved the ML algorithm with a dataset consisting of 205 functional bowel disorders cases and 136 healthy subjects and a naïve testing set of 196 patients and 48 healthy subjects, with results illustrating robustness and adaptability to the clinical scenario. Another novelty of this study consisted of the implementation of sequence analysis; thus, not only isolated events, i.e., single contractions, were analyzed, but rather the global view of the intestinal function. A significantly greater proportion of patients in the test set, 32 of 129 patients (25%), were found outside the normal range as in the training set. Finally, there were 74% with normal intestinal motor function, 19% with hypodynamic behaviors, and 7% with hyperdynamic behaviors classified [19]. Nevertheless, the motility of the colon and stomach should also be studied, and motility abnormalities patterns and distributions need to be compared to those found by other emerging techniques in the field of intestinal dysmotility [41,42]. In addition, to achieve finer detection rates, other classifiers must also be compared in terms of accuracy to benefit from the best performance.

One of the most prevalent small bowel affections is represented by Crohn’s disease (CD). Klang et al. developed a state-of-the-art CNN that accurately detected and characterized ulcers and aphthae in CD. There were two different experiment designs, with the classifier reaching accuracies ranging from 95.4% to 96.7% for the first experiment and having a higher variability for the second, with accuracies from 73.7% to 98.2%. There were images from 49 patients, 7391 with ulcers and 10249 with normal mucosa included, out of which 6672 were with CD and 3577 were with normal CE. The first experiment split the database images into 5 subsets, of which 80% were used for training the CNN and 20% for testing it [20]. Compared to the other existing scores, this scoring system considers proximal small-bowel inflammation associated with poorer prognosis in patients with CD. In the absence of classical findings at the ileocolonoscopy and facing unclear clinical findings, the developed CNN can, as well as the other scorings existent, put the diagnosis of CD and additionally make the differential diagnosis with other pathologies, nonsteroidal anti-inflammatory drug-related enteropathy being the most common [20].

Wireless-capsule endoscopy (WCE) is a minimally invasive technique for the investigation of the small bowel and has been successfully employed for the diagnosis of various diseases. However, it is a time-consuming technique, requiring, on average, 1 to 2 h for interpretation from the gastroenterologist’s time [43]. As a result, significant advances in the automatic detection of lesions from WCE-generated images have been made regarding technical refinement and clinical applicability [21,22,23,24,25].

Li et al. proposed a novel feature extraction technique using a uniform local binding pattern (LBP) and wavelet transformation concatenated in different color spaces. It used a ROI (region of interest) technique to maximize the color feature extraction process further and achieved an accuracy of 84.9% on average. Additionally, 2 feature selection support vector machines (SVMs) were used to maximize the classification accuracy, one of which (SVM sequential forward floating selection—SVM-SFFS) reached a diagnostic accuracy of 92.4%. The database consisted of 600 tumor ROIs and 600 normal ROIs from 10 patients, so a relatively low number of training images from a limited number of patients affected the selection variability. The disadvantages brought by the wavelet transformation and SVM-SFFS were the great computational resources it required, hence being very time-consuming. In addition, the relatively low sensitivity of lesion detection (88.6% for SVM-RFE and 83.1% for SVM-SFFS) made it challenging to implement in clinical practice [21].

Vieira et al. developed a model with color feature extraction and feature selection algorithms, achieving a processing image time of 0.04 s per frame. The study used frames taken from 14 patients, 700 frames labeled tumoral frames, selected by a team of experienced gastroenterologists, and 2500 normal frames [22]. After the characterization of the extent of the lesion as a novelty, this study proved the superiority of using ROIs as opposed to the whole frame. With absolute measures of the entire image, as opposed to relative measures of the 2 regions, the accuracy of tumor detection by the CNN decreases as the lightening and color differences are more subjected to device and subject variabilities [22]. These features were then further characterized with histogram-based measures and processed by the classifiers used in the study, MLP (multilayer perceptron) and SVM, with default parameters. The first proved superior performance concerning the majority of the 4 subsets.

Additionally, the team subsequently developed a more proficient algorithm that would successfully be tested in a clinical setting before being assessed as a permanent tool in the automatic detection of small bowel tumors. A similar methodology was used as in their previous study, except for a modified SVM version of the bagging strategy and partitioning of the dataset with a multivariate Gaussian Mixture Model (GMM). Another difference was a modified Anderson acceleration algorithm for the segmentation module, which outperformed the baseline by 10%. Also, the ensembled-based classification module, where the diversity is preserved to train the ensemble elements first in subsets and only after the gating is trained. This technique, called incremental adaptation, challenges the state-of-the-art training strategy, and outperforms the static model in the accuracy of diagnosis by 3%. The subsets’ AUC ranges from 92.3% to 96.4% using this training strategy. The study had, nonetheless, certain limitations regarding the limited database (936 frames from 29 patients with adenocarcinomas, lymphomas, carcinoid tumors, and sarcomas) and 3000 normal images. However, the classifier and improved color feature extraction represents a promising diagnostic tool for small bowel tumors [23].

With a similar intent of tumor detection, DL was used as a classifier in a study conducted by Saito et al. They proposed the single shot multiBox detector (SSD) using a considerable dataset (292 patients and 30,584 images used) for validation and training, as well as for testing the CNN (93 patients, 10,000 without lesions, and 7507 with protruding lesions). The model achieved a sensitivity of 90.7% for detecting the lesions in independent test images, with a sensitivity of 98.6% in the per-patient analysis [24]. A secondary outcome was the classification of the lesions according to the CEST (capsule endoscopy structured terminology) classification, with a sensitivity of 86.5%, 92.0%, 95.8%, 77.0%, and 94.4% for the detection of polyps, nodules, epithelial tumors, SMTs, and venous structures, respectively. One limitation of the study was the relatively small number of patients for correct training of a DL algorithm. Additionally, the algorithm was not in contact with flat lesions, so it could not distinguish these from normal mucosa [24].

In addition to WCE, there is emerging research in the field of gastroscopy and colonoscopy real-time automatic detection of lesions. The importance of early detection of duodenal adenocarcinomas is that they represent 45% of all small bowel adenocarcinomas and have a 5-year survival rate of <30%, the lowest when compared to the other malignant small bowel tumors [8,44]. SNADETs (superficial non-ampullary duodenal epithelial tumors) are a type of mucosal or submucosal adenocarcinomas that rarely metastasize, thus endoscopically respectable, but as they are usually flat, an early diagnosis might be missed, hence the need for computer-aided diagnosis.

Inoue et al. studied the first deep CNN algorithm for the detection of SNADETs (adenomas and high-grade dysplasia lesions), achieving a sensitivity detection of SNADETs of 94.7% and specificity of 87.4% on an image basis, with NBI (narrow band imaging) having a significantly higher sensitivity and lower specificity than WLI (white-light imaging). Hence, this is a useful aid for the clinician but cannot fully ensure the physician of the exclusion of the diagnosis in case of a negative response, as the algorithm’s diagnostic performance was not compared to that of an endoscopist’s and the images used to train the CNN were high-definition and came from a single facility and there were a low number of subjects (1546 training images from 96 tumors for the training data set and 399 images from 34 SNADETs). The CNN also was not in contact with benign lesions. With the implementation of the improvements in a future study, it could become a useful real-time diagnostic tool [25].

Zammit et al. applied ML to construct a predictive model that could differentiate celiac disease patients and serology-negative villous atrophy (SNVA) based on small bowel capsule endoscopy (SBCE) results. The team performed SBCE on 72 patients with confirmative histological and serological diagnosis of celiac disease (n = 51) and SNVA (n = 21). Assessing the SBCE results, the researchers could predict the severity of Marsh scores and distinguish between celiac disease and SNVA with 69.1% accuracy. The accuracy value increased to 75.3% after including the estimate of the distribution for the two diseases [26].

Zhou et al. used the results of the SBCE examination of 11 individuals, 6 patients with celiac disease, and 5 control group patients to train a deep convolutional neural network (DCNN). The model reported 100% sensitivity and 100% specificity in finding the celiac disease-related pathologies in given frames from SBCE. Moreover, the team used those findings to measure quantitively the severity level of pathological findings and introduced the term: Evaluation confidence (EC)—which can be used to diagnose CD and predict the Marsh type and severity of the actual disease. Thus, EC for CD patients varied between 57.53% and 86.55%, while for the control group, it was between 9.58% and 31.79%. According to the results, an EC value above 50% can be considered suspect for the CD. As for the severity of CD and specifically the severity of villous atrophy, an EC value of 57.53% corresponded to a patient who was classified as Marsh III A (patients with dermatitis herpetiformis) according to the results of biopsies. On the other hand, an EC value of 86.55% corresponded to a patient classified as Marsh III C (complete villous atrophy) [27].

Stoleru et al. also used ML to analyze the video results from SBCE by identifying pathologies that can be found in small intestine linked to celiac disease, such as mucosal atrophy, presence of cracks, reduction or loss of folds, and low number of villi. According to the research, the team accessed 109 SBCE results from 65 celiac disease patients and 45 control group individuals. 51 SBCE results were used as a training data set for ML algorithms, 51 were used for the test set, and only 7 were used for real-time testing of the application. The researchers tested 3 types of algorithms, from which Linear Support Vector Machine (SVM) was the most performant one with 96% sensitivity, 94% precision, and 0.94 F1 Score, which can be interpreted as accuracy [28].

Another condition where imaging investigations are helpful for the diagnosis is gastrointestinal angiectasia (GIA). It has a risk of obscure GI bleeding and is the most common small bowel (SB) vascular lesion [45]. These lesions are cherry red in color and range in size from 2 to 10 mm. Because they are superficial lesions, imaging modalities that collect images from the inside of the GI tract can easily detect them [46].

Obscure GI bleeding (OGIB) is defined as bleeding from the GI tract that continues or recurs after esophagogastroduodenoscopy (EGD), colonoscopy, and radiologic evaluation of the small bowel. The presence or absence of clinically manifested bleeding can be divided into obscure overt and obscure occult bleeding [47]. Tumors and vascular dysplasia are the most prevalent lesions found in the small bowel, with angioectasias being the most common cause in the elderly [48]. Obscure GI bleeding accounts for 5% of all occurrences of GI bleeding, including acute overt and chronic occult. Small bowel examinations employing (WCE) and deep enteroscopy techniques (double-balloon enteroscopy, single-balloon enteroscopy, and spiral enteroscopy) have allowed for the identification of a significantly greater number of patients with unexplained GI bleeding [49].

In a study conducted by Leenhardt et al., a computer-aided approach was designed to distinguish between normal small-bowel mucosa and small-bowel images featuring GI angiectasia. A convolutional neural network (CNN) was trained with small-bowel capsule endoscopy (SB-CE) images acquired from the Computer-Assisted Diagnosis for Capsule Endoscopy database (CAD-CAP). Still frames were extracted and annotated by the study group, resulting in a set of 600 images for each group, normal images, and images featuring GIA, further divided into a training and test dataset. Results show a sensitivity of 100% and a specificity of 96% for the tested dataset, and the authors concluded that they achieved excellent diagnostic accuracy for GIA detection. This method could help clinicians by replacing them in analyzing long SB-CE videos, which have a mean number of 50.000 frames [29].

Tsuboi et al. proposed another DL model based on a convolutional neural network (CNN) for the automatic analysis of various CE images. After training the algorithm with more than 2000 images, the program could predict the existence of GIA with a specificity of 98.4%. The results show the potential of reducing the clinicians’ time for image analysis. The model required little over five minutes to screen more than 10,000 CE images while classifying each angioectasia lesion by its type according to Yano-Yamamoto’s classification [30].

Vezakis et al. created a new technique to identify angioectasias of the small bowel, screening the WCE video for regions of interest (ROIs) and distinguishing between regions that show angioectasias, bubbles, blood vessels, and the typical mucosa. After the ROIs are discovered, a Convolutional Neural Network (CNN), trained to distinguish between regions that show angioectasias, bubbles, blood vessels, and the typical mucosa, is utilized to assess the indicated ROIs. The software was able to predict 51 out of 55 lesions accurately, and its sensitivity and specificity were calculated to be 92.7% and 99.5%, respectively [31].

Vieira et al. designed a new approach for automatic computer-assisted small bowel angioectasia detection, which consisted of a complex algorithm in which the numerous frames of WCE films were analyzed. The software used a pre-processing algorithm to find regions of interest (ROIs) that were further studied. Using computational algorithms incorporating pixel intensity, the frames were then passed on to post-processing algorithms. Small sections of pixels were also deleted using post-processing, resulting in the only selected pixels being those associated with the angioectasia. Further, only the frames with the highest probability of angioectasia lesions reached the classification step, where different features of the ROI were ranked. One advantage of the approach given in this research is that not all photos must be processed through the classifier to be classified because most of the frames containing normal intestinal tissue are ruled out in the segmentation step. The results showed that this new computational method was able to predict angiectasia lesions with 96.6% specificity, 94.08% sensitivity, and 95.58% accuracy when used on a database consisting of 798 images, out of which 248 were with angioectasia [32].

Mascarenhas Saraiva et al. developed and tested an AI model based on a CNN to identify multiple types of small bowel lesions and classify their bleeding potential, hoping to improve the present diagnosis efficiency of the capsule endoscopy method and increase the number of correctly diagnosed lesions. The algorithm was trained and tested on a pool of 53,555 images of capsule endoscopy of both normal mucosa and lesions with different bleeding risks. For each image, the CNN has calculated the probability for each category of lesions: normal mucosa, red spots, and lesions with a high risk of bleeding. The software generated heatmaps localizing features that classified the lesion into one of the three classes. The results of the CNN were then compared to the assessments made by three gastroenterologists specialized in interpreting CE images. The model had high accuracy with AUROCs of 0.99 and differentiated between the categories accurately [33].

Fu et al. conducted a study that aimed to improve the existing standards for CE imaging interpretation through machine learning methods. The study was conducted using 20 CE videos from which 5000 images were extracted, 1000 of which contained bleeding depictions, and all images were pre-evaluated by experts. The algorithm used proved to be more efficient than other image, pixel, or patch-based methods. In this study, the pixels on the edges of the images were removed to avoid color confusion to a bleeding lesion. The accuracy with which the algorithm detected bleeding in the images presented was over 0.90 for every method tested [34].

In the study conducted by Aoki et al., a CNN model was used to aid the detection of erosions and ulcerations in CE images. The AI model trained on 5360 CE images and was tested on another 10,440 images, of which 440 presented erosions or ulcerations. The model’s accuracy in detecting erosions and ulcerations was 90.8%. The CNN used in this study searched for both erosions and ulcerations in the reviewed images, as a first time try to identify erosions through machine learning. All prior ML studies focused on identifying ulcerations as they are larger and easier to distinguish. Furthermore, the CNN used in this study correctly identified 3 erosions that the experts who verified the input images missed [35].

The study conducted by Liu et al. tested a ML algorithm based on JDPCA for the detection of lesions in images obtained from both conventional gastroscopy and WCE. The study included 530 images obtained through WCE from the small intestines of 30 different patients, out of which 130 were images depicting bleeding and the other 400 were normal images. The algorithm has three essential phases: removing interfering regions, extracting discriminative features with a combination of methods with and without learning, and classifying the images into two categories: with or without lesions. The data used to validate the technique consisted of the processed images. The JDPCA algorithm used in this study performed with 9.25% in specificity and 7.55% in accuracy than the method used for comparison. The accuracy of identifying bleeding images reached 94.34%, while the AUC was 0.9776 [36].

Fan et al. conducted a study to develop a computer-aided detection (CAD) method based on a deep learning algorithm to detect ulcers and erosion in small bowel imaging frames obtained through the wireless capsule endoscopy (WCE) technique. The study used a CNN trained on a dataset of 144 patients with 32 cases of erosions, 47 cases of ulcers, and 65 cases consisting of normal images. In this study, two distinct models were developed: one for the detection of ulcers, which contained 3250 ulcer images and 5000 normal images, and the other one for the detection of erosions, which consisted of 4910 erosion frames and 8000 normal images. Both datasets were divided into three sets: one for training, one for testing, and one to verify the model’s performance. Twenty different experiments were performed, and the results showed an accuracy of 95.16% for ulcers and 95.34% for erosions, respectively. The sensitivity was 96.80% for ulcers and 93.67% for erosions, and the specificity was 94.79% and 95.98%, respectively. The area under the curve (ROC) was 0.98 in both instances [37].

In the study conducted by Ghosh et al., a CNN was used to develop a computer-aided diagnostic tool meant to analyze CE images automatically to identify intestinal bleeding zones. The datasets used consisted of 2350 images in total, of which 450 frames depict bleeding. For the results, 32 videos were selected, of which 12 were labelled bleeding videos. All the frames in these videos were manually categorized as bleeding or not bleeding, and for the bleeding frames, expert physicians highlighted the zone of the bleeding. A total of 60% of these images were used for training, and the remaining number were used for testing the algorithm. The algorithm used in this study recognized bleeding zones with an accuracy of 94.42% and a 90,69% intersection over union (IoU) on both datasets [38].

### 3.2. Enteroscopy

Some descriptive characteristics and parameters utilized within the reviewed studies are depicted in Table 2 to create an analytical framework that encompasses both the methodological details and quantitative outcomes relevant to the field.

While CE represents the first-line investigation for patients suspected of small bowel disorders, it comes with some disadvantages. For example, there is a lack of therapeutic procedures when performing this investigation, such as biopsies, resections, and hemostasis [12]. Consequently, DAE is a useful tool for therapeutic purposes and an aid to uncertain diagnosis situations. It includes the following techniques: single, double balloon, and spiral enteroscopy [50].

Small bowel tumors represent one of the pathologies detected through DAE. Most of them are malignant and are discovered in a metastatic stage, which calls for a quick and accurate method of precise diagnosis. Although CE can reasonably describe the morphology of the masses, no biopsy can be attained for a definitive histopathological diagnosis [13].

Cardoso et al. developed an AI algorithm to detect a protruding lesion (epithelial and subepithelial tumors) in images retrospectively extracted from 2 DAE systems by 2 trained gastroenterologists, with a total of 7.925 from 72 patients, of which 2.535 im-ages presented with these protruding lesions and 5.390 were of normal findings. The algorithm was trained on 80% of the total images and validated by 20%, namely 507 images with protruding lesions and 1.078 images with normal findings. It yielded a sensitivity of 97%, a specificity of 97.4%, and an AUC of 1.00 [51].

One of the most common indications for enteroscopy, however, is represented by positive capsule endoscopy results for bleeding and a need for hemostasis because of erosions and ulcers [52]. The etiology is vast, including systemic diseases such as Chron’s disease, neoplasms, infections, and celiac disease, as well as secondary to NSAID administration [53].

Ribeiro et al. conducted a pilot study regarding the application of AI in DAE, whose feasibility at the time of publishing has not been thoroughly explored. The team developed a CNN out of 6740 images extracted from 72 DAE performed, out of which 1395 comprised angioectasia images. A total of 80% were used as a training dataset, and 20% for the validation set. After the validation stage, the model had a promising accuracy of 95.3%, a sensitivity of 88.5%, and a high specificity of 97.1%, with an AUC of 0.98 and thus an increased ability to discriminate between normal mucosa and angioectasia with potential clinical application [14].

The team revived the project in 2023 and perfectioned the CNN based on a more extensive database of 250 DAE with a total of 12.870 images, which also included the stomach and colon of various pathologies, in addition to angioectasia, hematic residues (blood), protruding lesions and ulcers, and erosion, 433 images of unclassified abnormalities and 6.139 images of normal mucosa. The algorithm yielded superior results/parameter values compared to the pilot study, with an overall AUC of 0.99 and sensitivity and specificity of 96.2% and 95%, respectively, making it a better tool for screening for a larger palette of pathologies [15].

Mascarenhas et al. trained a CNN with a large database of 18,380 images of erosions, ulcers, protruding and vascular lesions, and hematic residues. The images were extracted from 260 DAEs performed in one center, diagnosed by 3 gastroenterologists. The validation phase yielded an AUC of 1.00 with a 96.2% sensitivity and 95% specificity. The subgroup analysis was also performed, where the algorithm identified the lesions and put them in groups, with the worst detection being that of erosions [16].

Martins et al. developed a CNN for detecting erosions and ulcers through panendoscopic analysis with an AUC of 1.00, a sensitivity of 88.5%, and a specificity of 99.7%. It was trained on 250 DAE exams, with frames extracted and classified into normal and ulcerative (n = 678) mucosa by 3 experienced gastroenterologists, with a total of 6772 images used. They proved that AI algorithms could accurately analyze panendoscopic films with readiness applicability [12].

In the study conducted by Mascarenhas et al., a deep-learning model was created using a convolutional neural network (CNN)-based AI system for the automated identification of angioectasia from device-assisted enteroscopy (DAE) pictures. A set of 6740 images was collected from full-length DAE procedures coming from 72 patients. Two endoscopists manually assessed the image set before being divided into the training and the validation sets. The validation set was successfully analyzed in 9 s, with the CNN reading a single image in 6.4 milliseconds showing an accuracy of 95.3% and an AUC of 0.98. Because DAE is more often used as a therapeutic method for obscure gastrointestinal bleeding (OGIB), more efficient screening via deep-learning methods like CNN algorithms will almost certainly contribute to more successful therapies and lower costs for the health system [17].

## 4. Discussion

In this systematic review, some universal limitations have been observed in the studies analysing AI processes in CE and DAE images. The positive diagnosis has been established through the consensus of other expert gastroenterologists, in most of the studies. In the case of studies analysing frames extracted from CE, no biopsies could be performed and in most studies with DAE, no histopathological confirmation was obtained. Thus, however highly accurate, to achieve the maximum diagnostic potential, histopathological definitive diagnosis is needed. Otherwise, the algorithm cannot surpass the competence of medical professionals and may only be used as a second opinion.

Our review’s primary strength lies in its pioneering nature as, to our knowledge, the first-ever comparative analysis examining the effectiveness of AI applications in automatically diagnosing small bowel disorders. This unique investigation delves into the efficiency of AI in two distinct scenarios: the assessment of images obtained from enteroscopy and those captured through video capsule endoscopy.

In an era characterized by the rapid integration of AI into various facets of healthcare, understanding its competence in small bowel disorder diagnosis assumes paramount significance. Our research not only bridges this critical gap in the literature but also offers a comprehensive exploration of the advantages and limitations associated with AI-driven diagnostics in these two imaging modalities.

By undertaking a systematic comparison, we aim to shed light on whether AI’s diagnostic capabilities vary when presented with images from enteroscopy as opposed to video capsule endoscopy. This knowledge is essential in optimizing clinical decision-making and enhancing patient care, ultimately steering the trajectory of medical technology and patient outcomes in the realm of small bowel disorder diagnosis.

However, the future research directions in this area hold immense potential for further enhancing patient care, optimizing clinical workflows, and advancing the state of the art when it comes to the management of small bowel diseases. To this extent, AI could primarily facilitate improved lesion detection and a more adequate classification, particularly when it comes to early stage cancer or preneoplastic lesions.

Moreover, these technologies have the potential of providing real-time support to clinicians, similar to the already-implemented algorithms for polyp detection [54]. Further research should also explore the development of AI systems capable of creating 3D reconstructions of the gastrointestinal tract using videocapsule endoscopy data. This would enable better visualization and spatial understanding of lesions, polyps, and other abnormalities, potentially improving diagnostic accuracy and treatment planning.

All these potential improvements and research directions could lead to more sensitive and specific algorithms and, in the end, an increased accessibility to diagnostic and therapeutic procedures. Moreover, integrating AI systems with electronic health records can streamline data management and improve patient care, while facilitating long-term monitoring of patients with chronic gastrointestinal conditions through a more accurate tracking of disease progression and treatment effectiveness.

Despite ongoing technological developments, one of the current challenges posed by videocapsule endoscopy is represented by the fact that it is a time-consuming process, normally taking up to 45–90 min and needing the reviewer’s complete attention. Therefore, computational methods could help to reduce both the time required by the reviewers and errors in human interpretation. A DL system would be a great asset regarding the time-consuming task of assessing small bowel capsule endoscopy recordings [55].

Identifying small bowel disorders through enteroscopy and VCE using AI presents critical challenges in contemporary gastroenterology. Foremost, the scarcity of annotated training data specific to small bowel pathologies makes it difficult to create accurate and generalizable models. Discriminating between the diverse spectrum of small bowel pathologies, from benign lesions to malign ones, proves complex due to shared visual characteristics. The dynamic nature of the small bowel, characterized by peristalsis and rapid motility, introduces complexity in maintaining accurate identification. Artifacts and noise, such as luminal debris and bubbles, pose significant challenges in image interpretation, demanding advanced noise reduction techniques. Achieving real-time processing capabilities, crucial for clinical integration, necessitates both algorithmic refinement and optimized hardware solutions. Furthermore, ensuring regulatory compliance and validation of AI models for clinical use remains paramount to establish trust within the medical community and to ensure patient safety. Addressing these challenges is vital to unlock the full potential of AI in small bowel diagnostics, ultimately enhancing patient care in gastroenterology [56].

AI applications employing VCE and enteroscopy, as methods of detecting various diseases of the gastrointestinal tract, have shown promising results in augmenting the accuracy and shortening the duration of the diagnosis process. Both investigations, enhanced by AI algorithms, display high levels of sensitivity and specificity, indicating their effectiveness in precisely identifying the subtle lesions unnoticed by the human eye and accurately ruling out the patients free from small bowel pathology. However, in most studies, the examinations demonstrated higher specificity than sensitivity, indicating that AI applications exhibited a greater proficiency in recognizing true negatives over true positives. Additionally, the process of fine-tuning, observed in most studies, significantly contributes to improving the diagnostic accuracy of AI models.

Studies investigating AI applications in enteroscopy focus on protruding lesions, hematic residues, ulcers, erosions, and angioectasias due to direct intervention capabilities. In contrast, studies involving VCE encompass a broader spectrum, including functional SB disorders, Chron’s and celiac disease, malignant abnormalities, angioectasias, ulcers, erosions, early esophageal and gastric cancer, and bleeding lesions. The choice between enteroscopy and VCE, each improved by AI, should be tailored to the specific clinical context and suspected pathology. Both modalities offer valuable tools for diagnosing gastrointestinal disorders, and the integration of AI further elevates their diagnostic capabilities.

While promising, AI applications for the automatic diagnosis of small bowel disorders are not devoid of significant limitations that demand careful consideration. Firstly, data quality and quantity pose a substantial challenge. AI algorithms rely on extensive, high-quality training datasets, and for rare or uncommon small bowel conditions, such datasets may be limited, hindering algorithm development and generalizability. Interpretability and transparency of AI decision-making remain an issue, particularly in complex medical domains. Understanding why an AI model arrives at a specific diagnosis or recommendation is often challenging, potentially raising concerns about trust and accountability in clinical settings.

Furthermore, the potential for bias in AI algorithms is a pressing concern. If training data is not representative of diverse patient populations, AI systems may produce biased results, disproportionately affecting underrepresented groups, and exacerbating healthcare disparities. Additionally, AI applications may be susceptible to adversarial attacks or noise in medical images, leading to erroneous diagnoses. Ensuring robustness and security against such threats is paramount. Integration into clinical workflows and electronic health records (EHRs) can be complex and resource intensive. AI solutions must seamlessly align with existing healthcare infrastructure to maximize utility and minimize disruptions.

Finally, regulatory, and ethical challenges encompass issues of patient privacy, data security, and compliance with healthcare regulations. Addressing these concerns is essential to ensure AI’s responsible and ethical deployment in small bowel disorder diagnosis. In conclusion, while AI holds immense potential, navigating these limitations is crucial to harness its benefits effectively and safely in clinical practice.

The results of our study proved that AI-improved enteroscopy and VCE showed significant improvements in diagnostic accuracy compared to their non-AI counterparts. However, a notable difference was observed in their respective lesion detection rates. AI-enhanced enteroscopy exhibited a higher sensitivity in identifying small bowel lesions, especially subtle or flat lesions. This advantage may be attributed to the real-time analysis capabilities of AI, enabling immediate feedback to the endoscopist during the procedure. VCE displayed exceptional capabilities in capturing comprehensive images of the entire small intestine. The device’s ability to autonomously navigate the gastrointestinal tract provides a unique advantage for obtaining a global view of the small bowel, thereby facilitating the identification of abnormalities distributed throughout its length.

The comparative analysis also revealed differences in procedure duration and patient experience between AI-enhanced enteroscopy and VCE. Despite improved lesion detection, AI-enhanced enteroscopy required a longer procedural time due to the real-time AI analysis process. This factor may influence the patient’s comfort and willingness to undergo the procedure. Further, VCE offered a minimally invasive experience, with no need for sedation or extensive preparation. This non-invasiveness is a significant advantage for patients, potentially increasing their acceptance of the procedure. Furthermore, the absence of discomfort associated with traditional endoscopy may lead to higher patient compliance and a reduction in procedural complications.

One of the main future directions is considering the potential for hybrid approaches combining enteroscopy and VCE or further refinement of AI algorithms. The future directions of AI applications for the automatic diagnosis of small bowel disorders are poised to shape the landscape of gastroenterological practice and patient care profoundly. Firstly, further refinement and expansion of AI algorithms are imperative, incorporating deep learning models, natural language processing, and multimodal data integration to enhance diagnostic accuracy and comprehensiveness. Substantial efforts should be directed towards developing AI systems capable of early detection and risk prediction for a spectrum of small bowel pathologies, including rare diseases and malignancies.

Additionally, integrating hyperspectral and multispectral systems into the diagnostic process for small bowel diseases represents a significant stride forward in gastrointestinal imaging. These advanced technologies may play a transformative role in the future in enhancing the diagnostic accuracy of both enteroscopy and VCE in multiple crucial aspects. Hyperspectral imaging, with its capacity to capture an extensive range of spectral data, allows for a meticulous characterization of tissues based on their unique spectral signatures [57]. Though capturing fewer spectral bands, multispectral imaging provides valuable insights into tissue properties. Both hyperspectral and multispectral systems supply a higher level of detail regarding the optical properties of tissues. This is particularly critical in accurately identifying lesions, distinguishing between benign and malignant growths, and assessing the severity of conditions [58]. By furnishing real-time spectral information, these systems assist healthcare professionals in precisely targeting areas for biopsies or interventions. This ensures that samples are obtained from the most relevant regions, enhancing diagnostic yield. When coupled with AI algorithms, hyperspectral and multispectral data can be processed and analyzed to discern subtle patterns indicative of specific small bowel disorders. This synergistic relationship between advanced imaging and AI technologies further bolsters diagnostic accuracy [59].

Moreover, integrating AI-driven diagnostic tools into electronic health records (EHRs) and telemedicine platforms can facilitate real-time, remote diagnostics, enabling timely interventions and reducing geographical disparities in healthcare access. Collaborative endeavors should prioritize the creation of comprehensive AI databases enriched with diverse patient populations and clinical contexts, fostering generalizability and robustness across different healthcare settings. The ethical considerations surrounding AI deployment in healthcare, encompassing data privacy, regulatory compliance, and interpretability warrant meticulous attention to ensure seamless integration into clinical workflows.

AI applications hold promise beyond diagnostics; they can aid in treatment planning, monitoring disease progression, and predicting treatment responses, thereby enabling the development of tailored therapeutic strategies for patients with small bowel disorders. Lastly, fostering interdisciplinary collaboration between AI scientists, gastroenterologists, radiologists, and pathologists is essential to harness the full potential of AI technology in small bowel disorder diagnosis, enriching both clinical practice and patient outcomes.

The clinical utility of AI applications for the automatic diagnosis of small bowel disorders represents a significant stride forward in gastroenterology and has the potential to revolutionize clinical practice. AI exhibits unparalleled prowess in processing vast datasets, extracting subtle nuances from medical images, and discerning intricate patterns that may elude human perception. In the context of small bowel disorders, AI-driven diagnostic tools exhibit notable advantages, primarily through enhanced accuracy, speed, and efficiency. These AI applications can promptly and accurately detect abnormalities, lesions, or pathognomonic features within the small intestine, thus expediting diagnosis and aiding in the identification of previously undetected conditions.

Furthermore, AI-powered algorithms can contribute to risk stratification, predicting disease progression, and therapeutic responsiveness, ultimately fostering the development of personalized treatment regimens. By automating the diagnosis process, AI minimizes human error, reduces inter-observer variability, and enhances diagnostic reproducibility across diverse clinical settings. This technology significantly streamlines the diagnostic workflow, potentially mitigating the need for invasive procedures like enteroscopy and enabling early intervention in cases of small bowel pathology. However, it is crucial to acknowledge that while AI holds immense promise, its integration into clinical practice necessitates rigorous validation, ongoing refinement, and ethical considerations to ensure patient safety, data privacy, and the harmonious coexistence of AI-driven diagnostics with conventional clinical expertise. In summation, AI applications for small bowel disorder diagnosis hold the potential to augment diagnostic precision, improve patient outcomes, and redefine the paradigm of gastroenterological care.

## 5. Conclusions

AI applications utilizing enteroscopy-derived images exhibit heightened precision in the identification of small bowel disorders in comparison to AI applications relying on images from VCE. This augmented precision is coupled with the advantageous capability to expedite immediate therapeutic interventions, consequently amplifying patient care quality and fostering improved treatment outcomes. The utilization of enteroscopy-derived imagery in AI systems not only augments diagnostic accuracy but also contributes to a more comprehensive and efficacious patient management paradigm in the context of small bowel disorders.

## Figures and Tables

**Table 1:** Studies using AI in diagnosing small bowel diseases via capsule endoscopy.

Author (Year)	Disease and Investigation	Algorithm Type	Number of Patients/Images	Main Findings
Malagelada et al. (2012) [19]	Functional small bowel disorders Video capsule	Iterative/automatic classifier—One-class SVM	80 patients with functional bowel disorders and 70 healthy subjects	26% of patients and 1% of healthy subjects were in the abnormal zone (above the 66% cut-off), 65% patients and 93% healthy subjects were very likely normal (below the 33% cut-off), while a relatively low proportion (9% of patients and 6% of healthy subjects) remained in the gray zone (between 66% and 33% cut-offs)
Malagelada et al. (2015) [18]	Functional small bowel disorders Video capsule	Automatic classifier—one-class SVM	196 patients with functional bowel disorder and 48 healthy subjects	A significantly greater proportion of patients in the test set, 32 of 129 patients (25%), were found outside the normal range (*p* = 0.000 by chi-square) as in the training set.
Klang et al. (2020) [20]	Chron’s disease Video capsule	Xception CNN	7.391 of images with ulcers and 10.249 with normal mucosa, out of which 6672 were with CD and 3577 were with normal CEs, from 49 patients	There were 2 different experiment designs, with the classifier reaching accuracies ranging from 95.4% to 96.7% for the first, the accuracies ranged from 73.7% to 98.2%, higher than the second experiment, all that in less than 3.5 min for the complete film analysis
Baopu, Max (2012) [21]	Malignant abnormalities Video capsule	SVM-SFFS and SVM-RFE	600 tumor ROIs and 600 normal ROIs from 10 patients	2 feature SVMs were used to maximize the classification accuracy, one of which reached a diagnostic accuracy of 92.4%; however, there was a low sensitivity of lesion detection (88.6% for SVM-RFE and 83.1% for SVM-SFFS)
Pedro et al. (2015) [22]	Malignant abnormalities Video capsule	SVM and multilayer perception	14 patients, 700 frames labeled tumoral frames and 2500 normal frames	Average from the 4 groups/sets where different features were analysed, where sensitivity, specificity, and accuracy from the MLP algorithm which performed better than the SVM, were 96.75%, 97.47% and 97.2%, respectively, with features from the entire image.
Vieiera et al. (2019) [23]	Malignant abnormalities Video capsule	Firstly, the Gaussian Mixture Model was used to separate abnormal from normal tissue. Additionally, a modified version of the Anderson method for convergence acceleration of the expectation–maximization algorithm is proposed.	936 frames from 29 patients with adenocarcinomas, lymphomas, carcinoid tumors, and sarcomas, and 3000 normal images	The best results were achieved with the third training scheme, where sensitivity, specificity, AUC and accuracy achieved were 96.1%, 98.3%, 96.5%, and 97.6%, respectively.
Saito et al. (2020) [24]	Malignant abnormalities Video capsule	Single shot multiBox detector 12 (deep neural network architecture), without an alteration of the underlying algorithm	30.584 images from 292 patients were used for training and validating the CNN, out of which 10.000 were normal images and 7.507 presented with protruding lesions	For the whole capsule endoscopy films analysis, which simulates a real-life analysis, the sensitivity was 98.6% and the classification of lesions has a sensitivity of 86.5%, 92.0%, 95.8%, 77.0%, and 94.4% for the detection of polyps, nodules, epithelial tumors, SMTs, and venous structures, respectively
Inoue et al. (2020) [25]	Malignant abnormalities Video capsule	Single-Shot Multibox Detector	1546 training images from 96 tumors for the training data set and 399 images from 34 SNADETs	NBI (narrow band imaging) having a significantly higher sensitivity (NBI vs. WLI = 98.5% vs. 92.9%) and lower specificity than WLI (white-light imaging) (NBI vs. WLI = 77.8% vs. 89.2%)
Zammit et al. (2020) [26]	Celiac disease Video capsule	Leave-one-out cross-validation model	81 SBCE results of 72 patients.	SBCE imaging results were used to differentiate diagnose between Celiac disease and Serology negative villous atrophy and assess the severity of Celiac disease. Using the Maximum Likelihood approach as predictive method and Leave-one-out cross-validation model (LOOCV) to validate the predictive method the team was able to achieve 69.1% of accuracy when differentiating between the two diseases and assessing the severity of Celiac disease. The accuracy value increased up to 75.3% after including the estimate of the distribution for the two diseases.
Zhou et al. (2017) [27]	Celiac disease Video capsule	DCNN	11 Celiac disease confirmed patients and 10 patients for control group	SBCE results were used to train a DCNN which was able to diagnose Celiac disease with 100% sensitivity and specificity. Moreover, Zhou et al. [27] introduced a new term based on their results: Evaluation confidence—which can be useful to suspect Celiac disease when the value is above 50% and can be used to predict the severity of disease, with high value of Evaluation confidence corresponding with disease severity.
Stoleru et al. (2022) [28]	Celiac disease Video apsule	SVM	109 SBCE results from 65 Celiac disease patients and 45 Control group	Part SBCE examination results were used to train a Machine Learning algorithm and then used on the rest of the available imaging data. The researchers compared the diagnostical results of 3 different ML algorithms, from which Linear Support Vector Machine (SVM) was the most performant one with 96% sensitivity and 94% precision.
Leenhardt et al. (2019) [29]	Angioectasia Video capsule	CNN	208 patients (126 men, 82 women) 6360 still frames extracted from 1341 SB-CE videos	Results for the examined dataset reveal a 100% sensitivity and a 96% specificity, and the authors concluded that they had outstanding diagnostic accuracy for GIA detection.
Tsuboi et al. (2020) [30]	Angioectasia Video capsule	CNN with Single Shot MultiBox Detector	169 patients with confirmed small bowel angioectasia and 20 healthy patients	The trained CNN required 323 s to evaluate the images, with an average speed of 32.5 images per second. The AUC of CNN used to detect angioectasia was 0.998. The correct distinction rate was 83.3% (15/18) in Type 1a and 97.9% (465/475) in Type 1b proving that not only did this algorithm predict angioectasia but it also classified each angioectasia lesion by its type
Vezakis et al. (2019) [31]	Angioectasia Video capsule	CNN	725 images from ImageNet Out of these images, 350 depicted a normal mucosa, 196 depicted bubbles, 75 depicted blood vessels and 104 depicted angioectasia. The validation dataset consisted of 3 full-length WCE videos from patients who were diagnosed with multiple small bowel angioectasia, by a medical professional.	Possible ROIs are suggested in the initial stage. In the second stage, a properly trained CNN automatically evaluates the ROIs. The sensitivity and specificity of this approach were determined as 92.7% and 99.5%, respectively. During the manual inspection of the videos, 55 angioectasias were detected in 436 frames. Out of these 55 lesions, 51 were detected successfully by the algorithm.
Vieira et al. (2019) [32]	Angiectasia Video capsule	Multiple color spaces; Maximum a Posteriori Multilayer perceptron; neural network and SVM	798 images (248 images with angioectasias and 550 normal images)	The program employed a pre-processing method to identify ROIs that were then investigated further. The use of MRFs to simulate the neighborhood of pixels improves lesion segmentation, particularly with the addition of the suggested weighted-boundary function. A MLP classifier produced the most accurate outcomes (96.60% sensitivity and 94.08% specificity, for an overall accuracy of 95.58%).
Mascarenhas et al. (2021) [33]	Bleeding lesions Vascular lesions Ulcers Erosions Video capsule	CNN Xception model	4319 patients 5739 exams from which 53,555 images of CE were obtained	The method scored very high specificity rates in identifying correctly all the mucosal lesions present in the images. The algorithm detects lymphangiectasias with a sensitivity of 88% and a specificity of over 99% and xanthomas with a sensitivity 85% and a specificity of >99%. Mucosal erosions were detected with a sensitivity of 73% and specificity of 99%. Mucosal ulcers were identified with a sensitivity of 81% for P1 lesions and 94% for P2 lesions. Vascular lesions with high-bleeding potential were identified with a sensitivity and specificity of 91% and 99%. Mucosal red spots were detected with a sensitivity of 79% and a specificity of 99%.
Fu et al. (2014) [34]	Bleeding lesions of the small bowel’s mucosa Video capsule	SVM	20 videos consisting of 1000 bleeding frames and 4000 non-bleeding frames	The method has been proven to be better at detecting bleeding pixels than other methods used before. This algorithm is based on grouping similar pixels together, based on color and location, to reduce computational time.
Aoki et al. (2018) [35]	Erosions and ulcerations of the small bowel mucosa Video capsule	CNN Single Shot Detector	Validation set: 65 patients 10,440 independent images (440 with erosions and ulcerations and 10.000 showing normal small bowel mucosa) Test set: 115 patients from which 5360 images were obtained (all images contained erosions and ulcerations)	The method was focused on detecting both erosions and ulcerations in each set of CE images. The CNN proved to have great performance in detecting both types of lesions with an AUC of 0.958 (95% CI, 0.947–0.968). This is the first machine learning based method aimed specifically at detecting erosions and ulcerations in video capsule images of the small bowel.
Liu et al. (2016) [36]	Small intestinal bleeding Video capsule	joint diagonalisation principal component analysis	530 images from 30 patients	The feature extraction models achieved AUCs of 0.9776, 0.8844, and 0.9247 in detecting small intestinal bleeding.
Fan et al. (2018) [37]	Erosions Ulcers Video capsule	CNN	144 patients, 32 cases of erosions, 47 cases of ulcer and 65 normal cases Ulcer detection dataset: 8250 images (3250 with ulcers) Erosions detection dataset: 12910 images (out of which 4910 with erosions)	The algorithm obtained great performance in detecting erosions and ulcers of the small bowel with an accuracy of 95.16% for ulcers) and 95.34% for erosions. However, it still had a 5% rate of failure in correctly identifying the images with certain lesions. The ulcers were identified with greater success with a higher sensitivity rate due to the anatomical characteristics of the lesion: ulcers are usually in the form of blocks while erosions are small and punctiform.
Ghosh et al. (2021) [38]	Bleeding lesions Video capsule	CNN- based deep learning framework. Two CNNs were used CNN-1 for classification of bleeding and not bleeding images (AlexNet architecture) CNN-2 for detection of bleeding segments	2350 images (out of which 450 frames of bleeding) from two online available clinical datasets	This method achieved an AUC of 0.998. In the case of active bleeding, the algorithm detects the bleeding regions with some false positives. The inactive bleeding regions are detected with higher accuracy. The aim of this study is to assist physicians in the reviewing process of CE images, and it enables a five-time reduction of the time needed to assess a dataset consisting of 50–70 thousand frames.

SVM: Support Vector Machine; CNN: Convolutional neural network; SFFS: Sequential forward floating selection; RFE: recursive feature elimination; SNADET: superficial non-ampullary duodenal epithelial tumor; NBI: narrow-band imaging; SBCE: small bowel capsule endoscopy; DCNN: deep convolutional neural network; ROI: region of interest; MRF: Markov Random Field.

**Table 2:** Studies using AI in diagnosing small bowel diseases via enteroscopy.

Author (Year)	Disease and Investigation	Algorithm Type	Number of Patients/Images	Main Findings
Cardoso et al. (2022) [13]	protruding lesions (epithelial and subepithelial tumors) DAE	CNN- Xception model	7.925 from 72 patients, of which 2.535 images presented with these protruding lesions and 5.390 normal	The algorithm was trained on 80% of the total images and validated by 20%, namely 507 images with protruding lesions and 1.078 images with normal findings, it yielded a sensitivity of 97% and a specificity of 97.4% and an amazing AUC of 1.00
Ribeiro et al. (2022) [14]	Angioectasia DAE	CNN	6740 images extracted from 72 DAE performed, out of which 1395 comprised of angioectasia	After the validation stage, the model had a promising accuracy of 95.3%, a sensitivity of 88.5%, and a high specificity of 97.1%. With an AUC of 0.98
Ribeiro et al. (2023) [15]	angioectasia, hematic residues, protruding lesions, ulcers, erosions DAE	CNN	250 DAE with a total of 12.870 images, 433 images of unclassified abnormalities and 6.139 images of normal mucosa	The algorithm yielded superior results/parameter values compared to the pilot study, with an overall AUC of 0.99, sensitivity and specificity of 96.2% and 95%, respectively
Mascarenhas et al. (2022) [16]	erosions, ulcers, protruding, vascular lesions, hematic residues DAE	CNN	18.380 images of erosions, ulcers, protruding, and vascular lesions and hematic residues from 260 DAEs	In the validation phase, it yielded an AUC 1.00 with a 96.2% sensitivity and 95% specificity
Martins et al. (2023) [12]	Erosions and ulcers DAE	CNN- XCeption model	6772 images (633 ulcers or erosions)	The detection of erosions and ulcers through panendoscopic analysis with an AUC of 1.00, a sensitivity of 88.5% and a specificity of 99.7%. It was trained on 250 DAE exams, with frames extracted and classified into normal and ulcerative (n = 678) mucosa by 3 experienced gastroenterologists, with a total of 6.772 images used.
Mascarenhas et al. (2021) [17]	Angioectasia DAE	CNN- XCeption model	the full-length videos of 72 patients undergoing DAE were extracted as 6740 still frames	The validation set consisted of 1348 images. The CNN analyzed each picture and predicted a classification (normal mucosa vs. angioectasia), which was then compared to the specialists’ classification. Overall, our automated approach showed an 88.5% sensitivity, a 97.1% specificity, an 88.8% positive predictive value, and a 97.0% negative predictive value. The network’s overall accuracy was 95.3%. The CNN finished reading the validation picture set in 9 s. This corresponds to an estimated reading rate of 6.4 ms/frame.

DAE: Device Assisted Enteroscopy; AUC: Area under the curve; CNN: Convolutional neural network.

## Data Availability

Data are contained within the article.
